# Granular Cell Tumor of the Toe: A Case Report

**DOI:** 10.1155/2010/184125

**Published:** 2010-08-24

**Authors:** Federico Tamborini, Mario Cherubino, Stefano Scamoni, Luigi A. Valdatta

**Affiliations:** Plastic Surgery Unit, University of Insubria, Circolo Hospital - Fondazione Macchi, Viale Borri 57, 21100 Varese, Italy

## Abstract

Granular cell tumor is a rare tumor of unknown etiology that more commonly affects the oral cavity but can also occur at other sites. The majorities of granular cell tumors are benign and present as a singular dermal nodule. We discuss a case of granular cell tumor of the fourth toe in a 54-year-old patient that was treated with conservative surgery, instead of amputation, and reconstruction with a dermal regeneration template.

## 1. Introduction

Granular cell tumor, also known as granular cell myoblastoma or Abrikossoff's tumor, is a rare tumor of unknown etiology that more commonly affects the oral cavity but can also occur at other sites. It develops between the second and sixth decades of life, more frequently among women and blacks. The neoplasm can affect all parts of the body. The head and neck areas are affected in 50% of cases and of these, 70% are located interorally (tongue, oral mucosa, hard palate) [1].

Granular cells tumor of the foot is rare, with only three tumors reported on the toe [2–4].

In general, it appears as a singular benign lesion; however there are rare cases that are malignant or multicentric forms [5–7]. The benign form shows polygonal cells with granular, eosinophilic cytoplasm and small nuclei. The malignant form, however, is associated with a high mitotic index and pleomorphic cellular tissue, tends to grow rapidly, and invades adjacent tissue [8].

The only examination that can confirm the clinical diagnosis is the histological examination. The treatment for Abrikossoff's tumor is surgery [9].

## 2. Case Report

**Figure 1:**
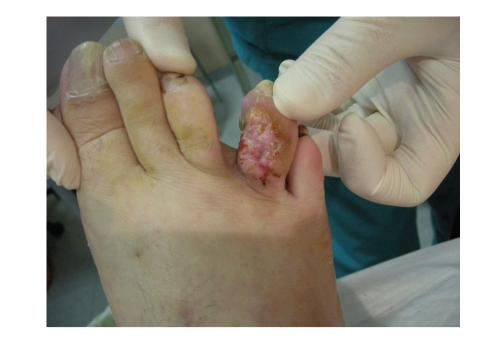
Granular cell tumor of the forth toe of the right foot.

A 54-year-old patient, with no prior history of skin cancer, was examined in October 2008 with a yellowish, hyperkeratotic neoformation causing pain, affecting the forth toe of the right foot (Figure 1); no local or popliteal lymphadenopathy was noted. A bioptic incisional examination was carried out, and the histological medical report found “granular cell tumor”. 

**Figure 2:**
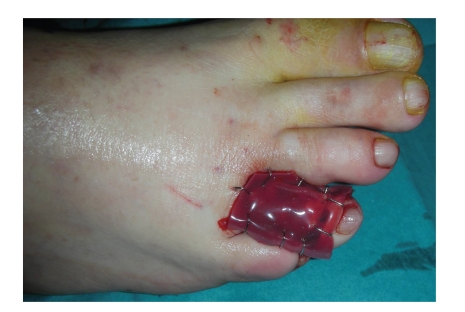
Immediate postop: reconstruction of the forth digit with the dermal regeneration template (Integra).

**Figure 3:**
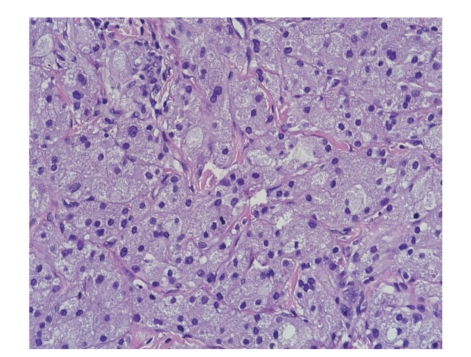
Histologic examination (200X).

The patient underwent surgery in our Unit to remove the neoformation. Intraoperative histologic examination of the resected specimen showed that the lesion had been totally excised. The toe was reconstructed with a dermal regeneration template made of cross-linked bovine collagen and chondroitin-6-sulfate (Figure 2) (Integra, Integra Life Sciences Corporation, Plainsboro, NJ). The final histological examination confirmed that the neoformation presents the histological characters of granular cell tumor (Figure 3). The tumor showed polygonal cells with abundant, eosinophil, and granular cytoplasm and small, uniform, hyperchromatic, and central nuclei. Immunohistochemical studies demonstrated that the tumor cells were S-100 protein and neuron-specific enolase (NSE) positive.

**Figure 4:**
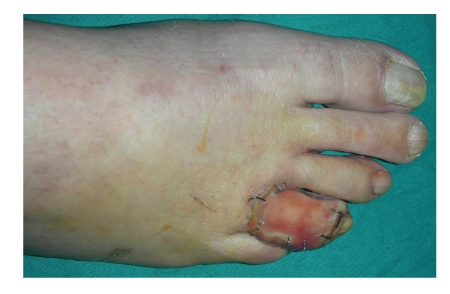
Postop at three weeks.

**Figure 5:**
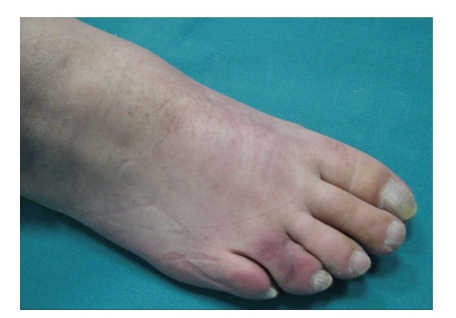
Clinical follow-up at one year.

Three weeks after surgery the silicone layer of the template was removed, and the toe healed with reepithelization in a month (Figure 4). No adjunctive surgery was necessary. At the postoperative clinical checkup, no complications were evident. The clinical followups carried out 6 months and one year after the operation have shown no relapse of the disease and complete resolution of the pain (Figure 5).

## 3. Discussion

Granular cell tumor, otherwise known as Abrikossoff's tumor, most often presents as stable or slow growing, benign, and solitary tumor less than 3 cm in diameter. Two-thirds of cases are reported in women, and two-thirds of cases are reported in black persons. It most commonly occurs between the fourth and sixth decades of life. The neoformation can affect all parts of the body with the highest concentration in areas with highest concentration of peripheral nerves. The head and neck areas are affected in 50% of cases and of these, 70% are located interorally (tongue, oral mucosa, and hard palate). The cutis and the subcutaneous tissue are affected in 30% of cases, the breasts in 15%, and the respiratory system in 10% of cases. Only 1% to 3% of all reported cases are malignant [1].

The malignancy of the neoformation is suggested by the rate of growth, the size (>4 cm), and the presence of necrotic and hemorrhagic areas whereas histologically it shows a high mitotic index and cellular and nuclear pleomorphism [8].

Both benign lesion such as hyperkeratotic or verrucous lesion and malign lesion such as squamous cell carcinoma or acral melanoma have to be considered as differential diagnosis.

Histologically, the benign tumor appears in no ulcerated nodular form, in varying dimensions from 2 to 5 cm. Microscopically the cells appear to show small, round, and central nuclei. The neoformation cells have a low mitotic index. The cytoplasm contains an abundant granular eosinophilic substance [1]. Typically the granules stain positive with periodic acid-Schiff (PAS) staining and are resistant to diastase digestion; they also stain with Sudan black B.

The origin of the granular cell tumor is controversial. The most substantiated hypothesis at present is that the lesion is a consequence of altered cellular metabolism of the Schwann cells. This theory is supported by the presence of the protein S-100, a marker regularly expressed in tumors of neural origin [10].

The treatment, exclusively surgical, consists of local excision of the neoformation. The case we report is uncommon: only three granular cell tumors have been reported on the toe; none of these were treated with conservative surgery and reconstructed with a dermal regeneration template. Amputation has been the surgical first choice.

In 15% of cases reappearance locally is possible in the incomplete excision of the neoformation [1]. The use of radiotherapy and chemotherapy is advisable only in treating the malignant forms of such tumors.

## 4. Conclusion

Granular cell tumor is a rare tumor of unknown etiology. Only three cases are documented in the toe, all treated with partial or total digital amputation. We reported a case of granular cell tumor of the forth toe in a 54-year-old patient that was treated with conservative surgery and reconstruction with a dermal regeneration template. The clinical followups have shown no relapse of the disease and complete resolution of the pain.

## References

[1] Becelli R, Perugini M, Gasparini G, Cassoni A, Fabiani F (2001). Abrikossoff’s tumor. *Journal of Craniofacial Surgery*.

[2] Stock D, McKee P, Donley B, Lakin R, Goldblum J, Howard M (2009). Granular cell tumor of the toe: a case report. *Journal of Foot and Ankle Surgery*.

[3] Abraham T, Jackson B, Davis L, Yu J, Peterson C (2007). Mohs surgical treatment of a granular cell tumor on the toe of a child. *Pediatric Dermatology*.

[4] Peters JS, Crowe MA (1998). Granular cell tumor of the toe. *Cutis*.

[5] Sargenti-Neto S, Brazão-Silva MT, do Nascimento Souza KC (2009). Multicentric granular cell tumor: report of a patient with oral and cutaneous lesions. *British Journal of Oral and Maxillofacial Surgery*.

[6] Hatta J, Yanagihara M, Hasei M, Abe S, Tanabe H, Mochizuki T (2009). Case of multiple cutaneous granular cell tumors. *Journal of Dermatology*.

[7] Gross VL, Lynfield Y (2002). Multiple cutaneous granular cell tumors: a case report and review of the literature. *Cutis*.

[8] López-Jornet P (2008). Granular cell tumor of the tongue. *The New York state dental journal*.

[9] Chilukuri S, Peterson SR, Goldberg LH (2004). Granular cell tumor of the heel treated with Mohs technique. *Dermatologic Surgery*.

[10] Behzatoğlu K, Bahadir B (2007). Malignant granular cell tumor with unusual histological features. *Pathology International*.

